# Supplementary material for: Mirror-gazing-induced dissociation impairs self-reported and implicit sense of agency: A causal investigation of dissociation and agency under controlled laboratory conditions
Source: PLoS One. 2026 Feb 19;21(2):e0341316. doi: 10.1371/journal.pone.0341316 (PMC12919786; doi:10.1371/journal.pone.0341316)
Supplement: S1 Text — (DOCX) [file pone.0341316.s001.docx]

The Mirror-Gazing Paradigm

A series of studies [1-3] showed that a standardized paradigm of mirror-gazing under low illumination produced intense experiences of depersonalization-derealization and dissociated identity. The mirror-gazing technique elicits depersonalization through visual distortions. Caputo referred to this effect as “a strange face illusion”, based on reports from participants that they saw their faces as distorted, or as those of another person, an animal, or a monster. Considering the strange face effect as a visual illusion, it could be explained by perceptual mechanisms such as the Troxler effect (the fading of objects presented in the periphery of the visual field) and processes of filling in perceptual data to compensate for the lack of sensory stimuli caused by prolonged staring [1, 4]. Alternatively, a psychodynamic interpretation of the illusion suggests that the visual apparitions represent different aspects of the self [5]. However, the cognitive explanations overlook the unique nature of face perception and self-identity, while the dynamic approach disregards perceptual processes. Neurocognitive models attempt to address both aspects. For example, one account suggested that the illusion stems from a disconnection between cortical areas that process sensory information about the bodily self, and those responsible for interpreting such information in relation to the psychological self [6]. Another account hypothesized that the cause is a disruption in advanced stages of visual processing, which involve recognizing facial features, binding them into a Gestalt, retrieving information about facial identity, and differentiating between self and other identities [7, 8]. The latter neurocognitive model has been given a clinical interpretation, with findings suggesting that the gradual disruption of neural network functioning during mirror and dyad gazing is associated with progressive dissociative processes [9]. According to these findings, the primary process is derealization, i.e., detached or altered perception of reality, which sets the ground to loss of sense of personhood (depersonalization), that might progress to the extreme case of fragmented identity.

References

1. Caputo GB. Apparitional experiences of new faces and dissociation of self-identity during mirror gazing. Percept Mot Skills. 2010;110(3):1125–38. <https://doi.org/10.2466/pms.110.C.1125-1138>
2. Caputo GB. Strange-face-in-the-mirror illusion. Perception. 2010;39(7):1007–8. <https://doi.org/10.1068/p6466>
3. Caputo GB, Lynn SJ, Houran J. Mirror-and eye-gazing: An integrative review of induced altered and anomalous experiences. Imagin Cogn Pers. 2021;40(4):418–57. <https://doi.org/10.1177/0276236620969632>
4. Mash J, Jenkinson PM, Dean CE, Laws KR. Strange face illusions: A systematic review and quality analysis. Conscious Cogn. 2023;109:103480. <https://doi.org/10.1016/j.concog.2023.103480>
5. Caputo GB, Bortolomasi M, Ferrucci R, Giacopuzzi M, Priori A, Zago S. Visual perception during mirror-gazing at one's own face in patients with depression. Sci World J. 2014;2014:946851. <https://doi.org/10.1155/2014/946851>
6. Derome M, Fonseca-Pedrero E, Badoud D, Morosan L, Van De Ville D, Lazeyras F, et al. Resting-state networks of adolescents experiencing depersonalization-like illusions: cross-sectional and longitudinal findings. Schizophr Bull. 2018;44(Suppl 2):S501–11. doi:10.1093/schbul/sby031
7. Caputo GB. Strange-face illusions during eye-to-eye gazing in dyads: specific effects on derealization, depersonalization and dissociative identity. J Trauma Dissociation. 2019;20(4):420–44. doi:10.1080/15299732.2019.1597807.
8. Caputo GB. Strange-face-in-the-mirror illusions: specific effects on derealization, depersonalization, and dissociative identity. *J Trauma Dissociation.* 2023;24(5):575–608. doi:10.1080/15299732.2023.2195394
9. Lange R, Caputo GB, Lynn SJ, Houran J. Mirror- and eye-gazing perceptions in advanced psychometric perspective: preliminary findings. *Psychol Conscious (Russell Sage Found).* 2022;9(3):230–42. doi:10.1037/cns0000328
