# Supplementary material for: Mirror-gazing-induced dissociation impairs self-reported and implicit sense of agency: A causal investigation of dissociation and agency under controlled laboratory conditions
Source: PLoS One. 2026 Feb 19;21(2):e0341316. doi: 10.1371/journal.pone.0341316 (PMC12919786; doi:10.1371/journal.pone.0341316)
Supplement: S2 Text — (DOCX) [file pone.0341316.s002.docx]

The Intentional Binding Task

We developed a version of the intentional binding task based on previous studies [1, 2]. Each trial started with a message guiding participants to be prepared. Then, an analog clock face, marked with conventional intervals (5, 10, 15, etc.; Fig. S5) was displayed. Instead of clock hands, a dot rotated around the center of the clock face at a constant speed of 2560 ms per cycle.

The task included four conditions: Baseline-Action, Agency-Action, Baseline-Outcome, and Agency-Outcome. Each condition consisted of 23 trials. All participants performed all conditions in a random order. The events and instructions for every condition are detailed in Table S6.

In the Agency-Action and Agency-Outcome conditions, participants were instructed to press the space bar whenever they wished, and a tone was heard 250 ms after the key press. On the next screen, an empty clock face was shown, and participants were asked to indicate either the time they heard the tone (in the Agency-Outcome condition) or the time they pressed the key (in the Agency-Action condition). The baseline conditions were created to assess participants’ time estimation at a baseline level, regardless of agentive action. Therefore, in the Baseline-Action condition, participants were asked to press the key and a tone did not follow their action. Then, as in the Baseline-Action condition, they were asked to indicate the time they pressed the key. In the Baseline-Outcome condition, no action was required but a tone was heard. Then, participants were asked to estimate the time they heard the tone. Upon the termination of conditions that included a tone, participants were asked to explicitly rate their sense of agency regarding tone production. The question: “To what extent did you feel that you generated the tones?” appeared on the screen, with a slider scale ranging from “not at all” (1) to “very much” (9).

The IB task yields implicit and explicit indices of the sense of agency. For the implicit ones, we calculated participants’ judgement errors for each trial, by subtracting the real time of the event from the estimated time of the event. Thus, in the Baseline-Action and Agency-Action conditions, we calculated the difference (in ms) between the real key press time and the estimated key press time. In the Baseline-Outcome and Agency-Outcome conditions, we subtracted the real tone time from the estimated tone time. For each participant, we calculated an average judgment error for each condition and also subtracted the average judgment error of each baseline condition from the average judgment error of the matching agency condition, creating measures named “Action binding” and “Outcome binding”. The binding measures were calculated to control for participants’ baseline timing judgments. Thus, each participant had four implicit indices: (1) Judgement error for the Agency-Action condition, (2) Judgement error for the Agency-Outcome condition, (3) Action binding, (4) Outcome binding. With all indices, greater deviations from the real timing indicate a higher sense of agency. The action indices are expected to be positive (since the action, when experienced as agentic, is perceived later than its actual timing and closer to the outcome), and a higher score in action indices marks a higher sense of agency. As for the outcome indices, the scores are expected to be negative (since an agentic outcome is perceived earlier than its actual timing), and thus a lower score represents a higher sense of agency.

The explicit indices calculated from the IB task were participants’ answers for the direct agency estimations of their perceived influence over the tone, with a higher score indicating a higher sense of agency. Their answers to the Agency-Action and Agency-Outcome conditions reflect a benign subjective sense of agency, whereas their answers to the Baseline-Outcome condition (in which they did not really control the tones in any way) reflect an illusory sense of agency.

References

1. Haggard P, Tsakiris M. The experience of agency: Feelings, judgments, and responsibility. Curr Dir Psychol Sci. 2009;18(4):242–6. <https://doi.org/10.1111/j.1467-8721.2009.01644.x>
2. Garaizar P, Cubillas CP, Matute H. A HTML5 open-source tool to conduct studies based on Libet's clock paradigm. Sci Rep. 2016;6:32689. <https://doi.org/10.1038/srep32689>
