# Supplementary material for: Mirror-gazing-induced dissociation impairs self-reported and implicit sense of agency: A causal investigation of dissociation and agency under controlled laboratory conditions
Source: PLoS One. 2026 Feb 19;21(2):e0341316. doi: 10.1371/journal.pone.0341316 (PMC12919786; doi:10.1371/journal.pone.0341316)
Supplement: S1 Table — (DOCX) [file pone.0341316.s003.docx]

**S1 Table**

*Descriptive Data of Study 1 Variables*

| **Measure** | **Condition** | **Time** | **Mean** | **SD** |
| --- | --- | --- | --- | --- |
| **State Depersonalization-Derealization** | Control (Video) | T1 | 4.25 | 4.98 |
|  |  | T2 | 7.38 | 6.70 |
|  |  | T3 | 5.34 | 5.73 |
|  | Mirror-Gazing | T1 | 4.30 | 5.91 |
|  |  | T2 | 8.40 | 7.86 |
|  |  | T3 | 5.67 | 6.11 |
|  | Mirror-Gazing and Suggestion | T1 | 3.97 | 6.23 |
|  |  | T2 | 8.69 | 8.92 |
|  |  | T3 | 3.94 | 6.15 |
| **State Absorption and Imaginative Involvement** | Control (Video) | T1 | 7.49 | 7.95 |
|  |  | T2 | 30.18 | 21.53 |
|  |  | T3 | 8.57 | 14.23 |
|  | Mirror-Gazing | T1 | 15.86 | 16.17 |
|  |  | T2 | 30.00 | 18.88 |
|  |  | T3 | 13.48 | 14.29 |
|  | Mirror-Gazing and Suggestion | T1 | 9.29 | 11.33 |
|  |  | T2 | 32.62 | 21.91 |
|  |  | T3 | 10.04 | 12.97 |
| **State Sense of Agency** | Control (Video) | T1 | 6.10 | 0.70 |
|  |  | T2 | 6.14 | 0.78 |
|  |  | T3 | 6.31 | 0.62 |
|  | Mirror-Gazing | T1 | 5.99 | 0.70 |
|  |  | T2 | 5.90 | 0.86 |
|  |  | T3 | 6.17 | 0.72 |
|  | Mirror-Gazing and Suggestion | T1 | 6.28 | 0.65 |
|  |  | T2 | 5.96 | 0.94 |
|  |  | T3 | 6.26 | 0.75 |
| **Trait Dissociation** † | Control (Video) | N/A | 14.40 | 8.77 |
|  | Mirror-Gazing | N/A | 18.37 | 9.95 |
|  | Mirror-Gazing and Suggestion | N/A | 18.33 | 13.00 |

*Note.* ^†^ The differences in trait dissociation between the study groups were not statistically significant.
