# Supplementary material for: Mirror-gazing-induced dissociation impairs self-reported and implicit sense of agency: A causal investigation of dissociation and agency under controlled laboratory conditions
Source: PLoS One. 2026 Feb 19;21(2):e0341316. doi: 10.1371/journal.pone.0341316 (PMC12919786; doi:10.1371/journal.pone.0341316)
Supplement: S2 Table — (DOCX) [file pone.0341316.s004.docx]

**S2 Table**

*Post-Hoc Contrasts Predicting Self-Reported State Dissociation and Sense of Agency, Controlling for Trait Dissociation (Study 1)*

| Predicting State Depersonalization-Derealization | | | | | | | |
| --- | --- | --- | --- | --- | --- | --- | --- |
| Level of trait dissociation | **Condition** | **Contrast** | **Estimate**  **[CI]** | **SE** | **t(df)** | ***p*** | **d** |
| Low Dissociation | V | T2 – T1 | 1.53  [-2.13, 5.18] | 1.62 | 0.95 (182) | .692 | 0.14 |
|  |  |  |  |  |  |  |  |
|  |  | T3 – T2 | -0.57  [-4.22, 3.08] | 1.62 | -0.35 (182) | .724 | -0.05 |
|  |  |  |  |  |  |  |  |
|  | MG | T2 – T1 | *7.27*  *[2.90, 11.63]* | *1.93* | *3.76 (182)* | *.001* | *0.56* |
|  |  |  |  |  |  |  |  |
|  |  | T3 – T2 | *-4.50*  *[-8.87, -0.13]* | *1.93* | *-2.33 (182)* | *.021* | *0.35* |
|  |  |  |  |  |  |  |  |
|  | MGS | T2 – T1 | 2.72  [-0.77, 6.21] | 1.54 | 1.76 (182) | .080 | 0.26 |
|  |  |  |  |  |  |  |  |
|  |  | T3 – T2 | *-3.98*  *[-7.47, -0.50]* | *1.54* | *-2.58 (182)* | *.022* | *-0.38* |
|  |  |  |  |  |  |  |  |
| Average Dissociation | V | T2 – T1 | *3.64*  *[0.87, 6.42]* | *1.23* | *2.97 (182)* | *.007* | *0.44* |
|  |  |  |  |  |  |  |  |
|  |  | T3 – T2 | *-2.50*  *[-5.28, 0.27]* | *1.23* | *-2.04 (182)* | *.043* | *-0.3* |
|  |  |  |  |  |  |  |  |
|  | MG | T2 – T1 | *4.45*  *[1.68, 7.21]* | *1.22* | *3.64 (182)* | *.001* | *0.54* |
|  |  |  |  |  |  |  |  |
|  |  | T3 – T2 | *-2.93*  *[-5.69, -0.16]* | *1.22* | *-2.39 (182)* | *.018* | *-0.35* |
|  |  |  |  |  |  |  |  |
|  | MGS | T2 – T1 | *4.27*  *[1.73, 6.82]* | *1.13* | *3.79 (182)* | *<.001* | *0.56* |
|  |  |  |  |  |  |  |  |
|  |  | T3 – T2 | *-4.51*  *[-7.06, -1.96]* | *1.13* | *-4.00 (182)* | *<.001* | *-0.59* |
|  |  |  |  |  |  |  |  |
| High Dissociation | V | T2 – T1 | *5.76*  *[0.84, 10.67]* | *2.17* | *2.65 (182)* | *.018* | *0.39* |
|  |  |  |  |  |  |  |  |
|  |  | T3 – T2 | *-4.44*  *[-9.35, 0.48]* | *2.17* | *-2.04 (182)* | *.043* | *-0.3* |
|  |  |  |  |  |  |  |  |
|  | MG | T2 – T1 | 1.62  [-2.19, 5.44] | 1.69 | 0.96 (182) | .675 | 0.14 |
|  |  |  |  |  |  |  |  |
|  |  | T3 – T2 | -1.35  [-5.17, 2.47] | 1.69 | -0.80 (182) | .675 | -0.12 |
|  |  |  |  |  |  |  |  |
|  | MGS | T2 – T1 | *5.83*  *[2.67, 8.99]* | *1.40* | *4.17 (182)* | *<.001* | *0.62* |
|  |  |  |  |  |  |  |  |
|  |  | T3 – T2 | *-5.04*  *[-8.20, -1.88]* | *1.40* | *-3.60 (182)* | *<.001* | *-0.53* |
|  |  |  |  |  |  |  |  |
| Predicting State Absorption | | | | | | | |
| Level of trait dissociation | **Condition** | **Contrast** | **Estimate**  **[CI]** | **SE** | **t(df)** | ***p*** | **d** |
| Low Dissociation | V | T2 – T1 | *14.22*  *[4.52, 23.91]* | *4.29* | *3.31 (182)* | *.002* | *0.49* |
|  |  |  |  |  |  |  |  |
|  |  | T3 – T2 | *-13.06*  *[-22.76, -3.37]* | *4.29* | *-3.04 (182)* | *.003* | *0.45* |
|  |  |  |  |  |  |  |  |
|  | MG | T2 – T1 | *24.12*  *[12.53, 35.72]* | *5.13* | *4.70 (182)* | *<.001* | *0.7* |
|  |  |  |  |  |  |  |  |
|  |  | T3 – T2 | *-17.18*  *[-28.77, -5.59]* | *5.13* | *-3.35 (182)* | *<.001* | *-0.5* |
|  |  |  |  |  |  |  |  |
|  | MGS | T2 – T1 | *17.58*  *[8.32, 26.84]* | *4.10* | *4.29 (182)* | *<.001* | *0.64* |
|  |  |  |  |  |  |  |  |
|  |  | T3 – T2 | *-16.59*  *[-25.85, 7.33]* | *4.10* | *-4.05 (182)* | *<.001* | *-0.6* |
|  |  |  |  |  |  |  |  |
| Average Dissociation | V | T2 – T1 | *25.43*  *[18.07, 32.80]* | *3.26* | *7.81 (182)* | *<.001* | *1.16* |
|  |  |  |  |  |  |  |  |
|  |  | T3 – T2 | *-24.37*  *[-31.73, -17.01]* | *3.26* | *-7.48 (182)* | *<.001* | *-1.11* |
|  |  |  |  |  |  |  |  |
|  | MG | T2 – T1 | *15.23*  *[7.90, 22.57]* | *3.25* | *4.69 (182)* | *<.001* | *0.7* |
|  |  |  |  |  |  |  |  |
|  |  | T3 – T2 | *-16.60*  *[-23.93, -9.26]* | *3.25* | *-5.11 (182)* | *<.001* | *0.76* |
|  |  |  |  |  |  |  |  |
|  | MGS | T2 – T1 | *22.70*  *[15.94, 29.46]* | *2.99* | *7.59 (182)* | *<.001* | *1.13* |
|  |  |  |  |  |  |  |  |
|  |  | T3 – T2 | *-22.08*  *[-28.85, -15.32]* | *2.99* | *-7.38 (182)* | *<.001* | *-1.09* |
|  |  |  |  |  |  |  |  |
| High Dissociation | V | T2 – T1 | *36.65*  *[23.60, 49.70]* | *5.77* | *6.35 (182)* | *<.001* | *0.86* |
|  |  |  |  |  |  |  |  |
|  |  | T3 – T2 | *-35.68*  *[-48.73, -22.63]* | *5.77* | *-6.18 (182)* | *<.001* | *0.92* |
|  |  |  |  |  |  |  |  |
|  | MG | T2 – T1 | 6.34  [-3.79, 16.47] | 4.48 | 1.41 (182) | .159 | 0.21 |
|  |  |  |  |  |  |  |  |
|  |  | T3 – T2 | *-16.01*  *[-26.14, -5.88]* | *4.48* | *-3.57 (182)* | *<.001* | *-0.53* |
|  |  |  |  |  |  |  |  |
|  | MGS | T2 – T1 | *27.82*  *[19.43, 36.21]* | *3.71* | *7.49 (182)* | *<.001* | *1.11* |
|  |  |  |  |  |  |  |  |
|  |  | T3 – T2 | *-27.58*  *[-35.97, -19.19]* | *3.71* | *-7.43 (182)* | *<.001* | *-1.1* |
|  |  |  |  |  |  |  |  |
| Predicting State Sense of Agency | | | | | | | |
| Level of trait dissociation | **Condition** | **Contrast** | **Estimate**  **[CI]** | **SE** | **t(df)** | ***p*** | **d** |
| Low Dissociation | V | T2 – T1 | 0.03  [-0.26, 0.32] | 0.13 | 0.22 (182) | .825 | 0.03 |
|  |  |  |  |  |  |  |  |
|  |  | T3 – T2 | 0.18  [-0.11, 0.47] | 0.13 | 1.41 (182) | .320 | 0.21 |
|  |  |  |  |  |  |  |  |
|  | MG | T2 – T1 | -0.29  [-0.63, 0.06] | 0.15 | -1.88 (182) | .125 | -0.28 |
|  |  |  |  |  |  |  |  |
|  |  | T3 – T2 | 0.24  [-0.11, 0.58] | 0.15 | 1.55 (182) | .125 | 0.23 |
|  |  |  |  |  |  |  |  |
|  | MGS | T2 – T1 | -0.05  [-0.33, 0.22] | 0.12 | -0.42 (182) | .675 | -0.06 |
|  |  |  |  |  |  |  |  |
|  |  | T3 – T2 | 0.17  [-0.10, 0.45] | 0.12 | 1.43 (182) | .307 | 0.21 |
|  |  |  |  |  |  |  |  |
| Average Dissociation | V | T2 – T1 | 0.04  [-0.18, 0.25] | 0.10 | 0.37 (182) | .715 | 0.05 |
|  |  |  |  |  |  |  |  |
|  |  | T3 – T2 | 0.16  [-0.05, 0.38] | 0.10 | 1.70 (182) | .182 | 0.25 |
|  |  |  |  |  |  |  |  |
|  | MG | T2 – T1 | -0.12  [-0.33, 0.10] | 0.10 | -1.20 (182) | .231 | -0.18 |
|  |  |  |  |  |  |  |  |
|  |  | T3 – T2 | 0.27  [0.05, 0.48] | 0.10 | 2.76 (182) | .013 | 0.41 |
|  |  |  |  |  |  |  |  |
|  | MGS | T2 – T1 | *-0.31*  *[-0.51, 0.10]* | *0.09* | *-3.44 (182)* | *.002* | *-0.51* |
|  |  |  |  |  |  |  |  |
|  |  | T3 – T2 | *0.27*  *[0.07, 0.48]* | *0.09* | *3.09 (182)* | *.002* | *-0.46* |
|  |  |  |  |  |  |  |  |
| High Dissociation | V | T2 – T1 | 0.04  [-0.35, 0.43] | 0.17 | 0.25 (182) | .804 | 0.04 |
|  |  |  |  |  |  |  |  |
|  |  | T3 – T2 | 0.15  [-0.24, 0.54] | 0.17 | 0.87 (182) | .774 | 0.13 |
|  |  |  |  |  |  |  |  |
|  | MG | T2 – T1 | 0.05  [-0.25, 0.36] | 0.13 | 0.41 (182) | .684 | 0.06 |
|  |  |  |  |  |  |  |  |
|  |  | T3 – T2 | 0.30  [-0.01, 0.60] | 0.13 | 2.22 (182) | .056 | 0.33 |
|  |  |  |  |  |  |  |  |
|  | MGS | T2 – T1 | *-0.56*  *[-0.81, 0.31]* | *0.11* | *-5.08 (182)* | *<.001* | *-0.75* |
|  |  |  |  |  |  |  |  |
|  |  | T3 – T2 | *0.38*  *[0.13, 0.62]* | *0.11* | *3.40 (182)* | *<.001* | *0.5* |
|  |  |  |  |  |  |  |  |

*Note*. *p*-values were adjusted using the Bonferroni Holm method. d = approximate Cohen’s d; V = Video watching control group; MG = mirror-gazing; MGS = mirror-gazing with suggestion; Statistically significant effects are italicized; Trait dissociation scores were assessed with the Dissociative Experiences Scale [1] ; Low trait dissociation = 6.22, average =17.04, high = 27.87.

References

1. Carlson EB, Putnam FW. An update on the Dissociative Experiences Scale. Dissociation. 1993;6(1):16–27.
