# Supplementary material for: Mirror-gazing-induced dissociation impairs self-reported and implicit sense of agency: A causal investigation of dissociation and agency under controlled laboratory conditions
Source: PLoS One. 2026 Feb 19;21(2):e0341316. doi: 10.1371/journal.pone.0341316 (PMC12919786; doi:10.1371/journal.pone.0341316)
Supplement: S3 Table — (DOCX) [file pone.0341316.s005.docx]

**S3 Table**

*The Intentional Binding Task: Instructions and Events per Condition*

| **Condition** | **Instruction given to participants at the beginning of the trial** | **Event** | **Question presented to participants at the end of the trial** | **Question presented to participants at the end of the block** |
| --- | --- | --- | --- | --- |
| Baseline-Action | Please press the space bar at a time of your choice | No event | Please indicate when did you press the key | No question was displayed |
| Agency-Action | Please press the space bar at a time of your choice | A tone was heard 250 ms after the key press | Please indicate when did you press the key | To what extent did you feel that you generated the tones? |
| Baseline-Outcome | The clock will start running soon, you don’t have to do anything | A tone was heard at a random time during clock rotation | Please indicate when did you hear the tone | To what extent did you feel that you generated the tones? |
| Agency-Outcome | Please press the space bar at a time of your choice | A tone was heard 250 ms after the key press | Please indicate when did you hear the tone | To what extent did you feel that you generated the tones? |
