# Supplementary material for: Mirror-gazing-induced dissociation impairs self-reported and implicit sense of agency: A causal investigation of dissociation and agency under controlled laboratory conditions
Source: PLoS One. 2026 Feb 19;21(2):e0341316. doi: 10.1371/journal.pone.0341316 (PMC12919786; doi:10.1371/journal.pone.0341316)
Supplement: S4 Table — (DOCX) [file pone.0341316.s006.docx]

**S4 Table**

*Descriptive Data of Study 2 Variables*

| **Dissociation Measure** | **Condition** | **Time** | **Mean** | **SD** |
| --- | --- | --- | --- | --- |
| State Depersonalization-Derealization | Video | T1 | 2.74 | 4.42 |
|  |  | T2 | 6.40 | 7.69 |
|  |  | T3 | 5.45 | 7.61 |
|  | Mirror-Gazing | T1 | 2.00 | 2.98 |
|  |  | T2 | 4.92 | 5.88 |
|  |  | T3 | 3.92 | 6.84 |
|  | Mirror-Gazing and Suggestion | T1 | 3.71 | 5.92 |
|  |  | T2 | 8.15 | 10.90 |
|  |  | T3 | 5.92 | 8.00 |
|  | Control | T1 | 3.24 | 5.23 |
|  |  | T2 | 4.06 | 4.38 |
|  |  | T3 | 4.12 | 5.42 |
| State Absorption | Video | T1 | 8.36 | 11.80 |
|  |  | T2 | 25.10 | 21.20 |
|  |  | T3 | 12.50 | 16.00 |
|  | Mirror-Gazing | T1 | 6.16 | 9.14 |
|  |  | T2 | 25.80 | 18.10 |
|  |  | T3 | 8.04 | 11.80 |
|  | Mirror-Gazing and Suggestion | T1 | 11.10 | 15.30 |
|  |  | T2 | 29.0 | 23.40 |
|  |  | T3 | 12.10 | 14.80 |
|  | Control | T1 | 8.11 | 12.60 |
|  |  | T2 | 14.60 | 16.90 |
|  |  | T3 | 18.20 | 23.20 |
| State Sense of Agency | Video | T1 | 6.12 | 0.75 |
|  |  | T2 | 6.14 | 0.85 |
|  |  | T3 | 6.01 | 0.97 |
|  | Mirror-Gazing | T1 | 6.36 | 0.67 |
|  |  | T2 | 6.18 | 0.83 |
|  |  | T3 | 6.18 | 0.99 |
|  | Mirror-Gazing and Suggestion | T1 | 6.06 | 0.94 |
|  |  | T2 | 5.88 | 1.08 |
|  |  | T3 | 5.94 | 1.09 |
|  | Control | T1 | 6.31 | 0.64 |
|  |  | T2 | 6.15 | 0.92 |
|  |  | T3 | 6.05 | 0.97 |
| Trait Dissociation^†^ | Video | N/A | 15.28 | 13.37 |
|  | Mirror-Gazing | N/A | 14.43 | 13.89 |
|  | Mirror-Gazing and Suggestion | N/A | 14.07 | 12.30 |
|  | Control | N/A | 13.72 | 11.08 |

*Note.* ^†^ The differences in trait dissociation between the study groups were not statistically significant.
