# Supplementary material for: Mirror-gazing-induced dissociation impairs self-reported and implicit sense of agency: A causal investigation of dissociation and agency under controlled laboratory conditions
Source: PLoS One. 2026 Feb 19;21(2):e0341316. doi: 10.1371/journal.pone.0341316 (PMC12919786; doi:10.1371/journal.pone.0341316)
Supplement: S5 Table — (DOCX) [file pone.0341316.s007.docx]

**S5 Table**

*Post-Hoc Contrasts Predicting Self-Reported State Dissociation and Sense of Agency, Controlling for Trait Dissociation (Study 2)*

| Predicting State Depersonalization-Derealization | | | | | | | |
| --- | --- | --- | --- | --- | --- | --- | --- |
| Level of trait dissociation | **Condition** | **Contrast** | **Estimate [CI]** | **SE** | **t(df)** | ***p*** | **d** |
| Low Dissociation | V | T2 – T1 | 1.74 [-0.64, 4.11] | 1.06 | 1.64 (382) | .203 | 0.17 |
|  |  | T3 – T2 | -1.71 [-4.09, 0.66] | 1.06 | -1.62  (382) | .203 | -0.17 |
|  | MG | T2 – T1 | 1.49 [-0.89, 3.87] | 1.06 | 1.41  (382) | .320 | 0.14 |
|  |  | T3 – T2 | -1.17 [-3.55, 1.21] | 1.06 | -1.11  (382) | .320 | -0.11 |
|  | MGS | T2 – T1 | -1.18 [-3.67, 1.30] | 1.10 | -1.07  (382) | .538 | -0.11 |
|  |  | T3 – T2 | 1.22 [-1.26, 3.71] | 1.10 | 1.11  (382) | .538 | 0.11 |
|  | C | T2 – T1 | 0.40 [-2.14, 2.93] | 1.13 | 0.36  (382) | .724 | 0.04 |
|  |  | T3 – T2 | -1.18 [-3.71, 1.35] | 1.13 | -1.05  (382) | .589 | -0.11 |
| Average Dissociation | V | T2 – T1 | *3.53 [1.87, 5.20]* | *0.74* | *4.77*  *(382)* | *<.001* | *0.49* |
|  |  | T3 – T2 | -0.99 [-2.66, 0.67] | 0.74 | -1.34  (382) | .181 | -0.14 |
|  | MG | T2 – T1 | *2.91 [1.16, 4.66]* | *0.78* | *3.75*  *(382)* | *<.001* | *0.38* |
|  |  | T3 – T2 | -1.00 [-2.75, 0.75] | 0.78 | -1.29  (382) | .199 | -0.13 |
|  | MGS | T2 – T1 | *4.59 [2.84, 6.34]* | *0.78* | *5.90*  *(382)* | *<.001* | *0.6* |
|  |  | T3 – T2 | *2.32 [-4.07, -0.57]* | *0.78* | *-2.98*  *(382)* | *.003* | *-0.3* |
|  | C | T2 – T1 | 0.84 [-0.87, 2.56] | 0.77 | 1.11  (382) | .539 | 0.11 |
|  |  | T3 – T2 | 0.13 [-1.59, 1.85] | 0.77 | 0.17  (382) | .865 | 0.02 |
| High Dissociation | V | T2 – T1 | *5.33 [3.11, 7.56]* | *0.99* | *5.40*  *(382)* | *<.001* | *0.55* |
|  |  | T3 – T2 | -0.28 [-2.50, 1.95] | 0.99 | -0.28  (382) | .781 | -0.03 |
|  | MG | T2 – T1 | *4.37 [1.97, 6.71]* | *1.05* | *4.12*  *(382)* | *<.001* | *0.42* |
|  |  | T3 – T2 | -0.83 [-3.20, 1.54] | 1.05 | -0.79  (382) | .430 | -0.08 |
|  | MGS | T2 – T1 | *10.36*  *[7.80, 12.91]* | *1.13* | *9.13*  *(382)* | *<.001* | *0.93* |
|  |  | T3 – T2 | *-5.86*  *[-8.42, -3.31]* | *1.13* | *-5.17*  *(382)* | *<.001* | *-0.53* |
|  | C | T2 – T1 | 1.29 [-1.40, 3.98] | 1.20 | 1.08  (382) | .458 | 0.11 |
|  |  | T3 – T2 | 1.44 [-1.25, 4.13] | 1.20 | 1.21  (382) | .458 | 0.12 |
| Predicting State Absorption | | | | | | | |
| Level of trait dissociation | **Condition** | **Contrast** | **Estimate [CI]** | **SE** | **t(df)** | ***p*** | **d** |
| Low Dissociation | V | T2 – T1 | *9.40 [2.62, 16.18]* | *3.01* | *3.12*  *(382)* | *.004* | *0.32* |
|  |  | T3 – T2 | *-8.59*  *[-15.37, -1.81]* | *3.01* | *-2.85*  *(382)* | *.005* | *-0.29* |
|  | MG | T2 – T1 | *13.87*  *[7.09, 20.65]* | *3.01* | *4.60*  *(382)* | *<.001* | *0.47* |
|  |  | T3 – T2 | *-14.64*  *[-21.42, -7.86]* | *3.01* | *-4.86*  *(382)* | *<.001* | *-0.5* |
|  | MGS | T2 – T1 | *10.76*  *[3.68, 17.85]* | *3.15* | *3.42*  *(382)* | *.001* | *0.35* |
|  |  | T3 – T2 | *-9.49*  *[-16.58 -2.40]* | *3.15* | *-3.01 (382)* | *.003* | *-0.31* |
|  | C | T2 – T1 | 3.05  [-4.17, 10.28] | 3.21 | 0.95  (382) | .342 | 0.1 |
|  |  | T3 – T2 | -5.33  [-12.55, 1.90] | 3.21 | -1.66  (382) | .196 | -0.17 |
| Average Dissociation | V | T2 – T1 | *16.23*  *[11.47, 20.99]* | *2.12* | *7.67 (382)* | *<.001* | *0.78* |
|  |  | T3 – T2 | *-12.32*  *[-17.08, -7.56]* | *2.12* | *-5.83 (382)* | *<.001* | *-0.6* |
|  | MG | T2 – T1 | *19.62*  *[14.63, 24.61]* | *2.22* | *8.85*  *(382)* | *<.001* | *0.91* |
|  |  | T3 – T2 | *-17.76*  *[-22.75, -12.77]* | *2.22* | *-8.01*  *(382)* | *<.001* | *-0.82* |
|  | MGS | T2 – T1 | *18.07*  *[13.08, 23.07]* | *2.22* | *8.15 (382)* | *<.001* | *0.83* |
|  |  | T3 – T2 | *-17.16*  *[-22.15, -12.17]* | *2.22* | *-7.74 (382)* | *<.001* | *-0.79* |
|  | C | T2 – T1 | *6.68*  *[1.78, 11.58]* | *2.18* | *3.07*  *(382)* | *.005* | *0.31* |
|  |  | T3 – T2 | 4.07  [-0.83, 8.97] | 2.18 | 1.87  (382) | .062 | 0.19 |
| High Dissociation | V | T2 – T1 | *23.06*  *[16.72, 29.40]* | *2.82* | *8.18 (382)* | *<.001* | *0.84* |
|  |  | T3 – T2 | *-16.06*  *[-22.40, -9.72]* | *2.82* | *-5.70 (382)* | *<.001* | *-0.58* |
|  | MG | T2 – T1 | *25.38*  *[18.61, 32.14]* | *3.00* | *8.45 (382)* | *<.001* | *0.86* |
|  |  | T3 – T2 | *-20.88*  *[-27.64, -14.11]* | *3.00* | *-6.95 (382)* | *<.001* | *-0.71* |
|  | MGS | T2 – T1 | *25.38*  *[18.10, 32.67]* | *3.24* | *7.85*  *(382)* | *<.001* | *0.8* |
|  |  | T3 – T2 | *-24.84*  *[-32.12, -17.55]* | *3.24* | *-7.68 (382)* | *<.001* | *-0.8* |
|  | C | T2 – T1 | *10.31*  *[2.63, 17.98]* | *3.41* | *3.02*  *(382)* | *.003* | *0.31* |
|  |  | T3 – T2 | *13.47*  *[5.80 21.15]* | *3.41* | *3.95*  *(382)* | *<.001* | *0.4* |
| Predicting State Sense of Agency | | | | | | | |
| Level of trait dissociation | **Condition** | **Contrast** | **Estimate [CI]** | **SE** | **t(df)** | ***p*** | **d** |
| Low Dissociation | V | T2 – T1 | 0.16 [-0.09, 0.43] | 0.11 | 1.47  (382) | .286 | 0.15 |
|  |  | T3 – T2 | 0.00 [-0.26, 0.25] | 0.11 | -0.03  (382) | .976 | 0.00 |
|  | MG | T2 – T1 | -0.04 [-0.30, 0.22] | 0.11 | -0.35  (382) | .730 | -0.04 |
|  |  | T3 – T2 | 0.21 [-0.05, 0.47] | 0.11 | 1.83  (382) | .136 | 0.19 |
|  | MGS | T2 – T1 | -0.12 [-0.38, 0.16] | 0.12 | -0.93  (382) | .390 | -0.10 |
|  |  | T3 – T2 | -0.16 [-0.42, 0.11] | 0.12 | -1.30  (382) | .390 | -0.13 |
|  | C | T2 – T1 | -0.04 [-0.32, 0.23] | 0.12 | -0.34  (382) | 1.00 | -0.03 |
|  |  | T3 – T2 | 0.06 [-0.22, 0.33] | 0.12 | 0.45  (382) | 1.00 | 0.05 |
| Average Dissociation | V | T2 – T1 | 0.03 [-0.15, 0.21] | 0.08 | 0.41  (382) | .684 | 0.04 |
|  |  | T3 – T2 | -0.12  [-0.30, 0.06] | 0.08 | -1.48  (382) | .282 | 0.15 |
|  | MG | T2 – T1 | -0.17 [-0.36, 0.02] | 0.08 | -2.03  (382) | .086 | -0.21 |
|  |  | T3 – T2 | 0.00 [-0.20, 0.18] | 0.08 | -0.07  (382) | .946 | 0.00 |
|  | MGS | T2 – T1 | -0.18 [-0.37, 0.01] | 0.08 | -2.13  (382) | .067 | -0.22 |
|  |  | T3 – T2 | 0.06 [-0.13, 0.25] | 0.08 | 0.69  (382) | .492 | 0.07 |
|  | C | T2 – T1 | -0.17 [-0.36, 0.01] | 0.08 | -2.08  (382) | .076 | -0.21 |
|  |  | T3 – T2 | -0.10 [-0.29, 0.08] | 0.08 | -1.24  (382) | .215 | -0.13 |
| High Dissociation | V | T2 – T1 | -0.10 [-0.34, 0.14] | 0.11 | -0.96  (382) | .339 | -0.10 |
|  |  | T3 – T2 | -0.23 [-0.47, 0.01] | 0.11 | -2.18  (382) | .060 | -0.22 |
|  | MG | T2 – T1 | *-0.30*  *[-0.56, -0.05]* | *0.11* | *-2.65*  *(382)* | *.017* | *-0.27* |
|  |  | T3 – T2 | -0.22 [-0.45, 0.04] | 0.11 | -1.94  (382) | .053 | -0.20 |
|  | MGS | T2 – T1 | -0.25 [-0.52, 0.03] | 0.12 | -2.02  (382) | .056 | -0.21 |
|  |  | T3 – T2 | 0.27 [-0.01, 0.58] | 0.12 | 2.21  (382) | .056 | 0.23 |
|  | C | T2 – T1 | *-0.31*  *[-0.59, -0.01]* | *0.13* | *-2.34*  *(382)* | *.040* | *-0.24* |
|  |  | T3 – T2 | *-0.26 [-0.55, 0.03]* | *0.13* | *-2.01*  *(382)* | *.045* | *-0.21* |

*Note*. *p*-values were adjusted using the Bonferroni Holm method. d = approximate Cohen’s d; V = Video-watching group; MG = mirror-gazing; MGS = mirror-gazing with suggestion; C = Control group (article). Statistically significant effects are italicized. Trait dissociation scores were assessed with the Dissociative Experiences Scale [1]; Low trait dissociation = 1.79, average = 14.39, high = 27.00.

References

1. Carlson EB, Putnam FW. An update on the Dissociative Experiences Scale. Dissociation. 1993;6(1):16–27.
