# Supplementary figures and images for: Mirror-gazing-induced dissociation impairs self-reported and implicit sense of agency: A causal investigation of dissociation and agency under controlled laboratory conditions
Source: PLoS One. 2026 Feb 19;21(2):e0341316. doi: 10.1371/journal.pone.0341316 (PMC12919786; doi:10.1371/journal.pone.0341316)

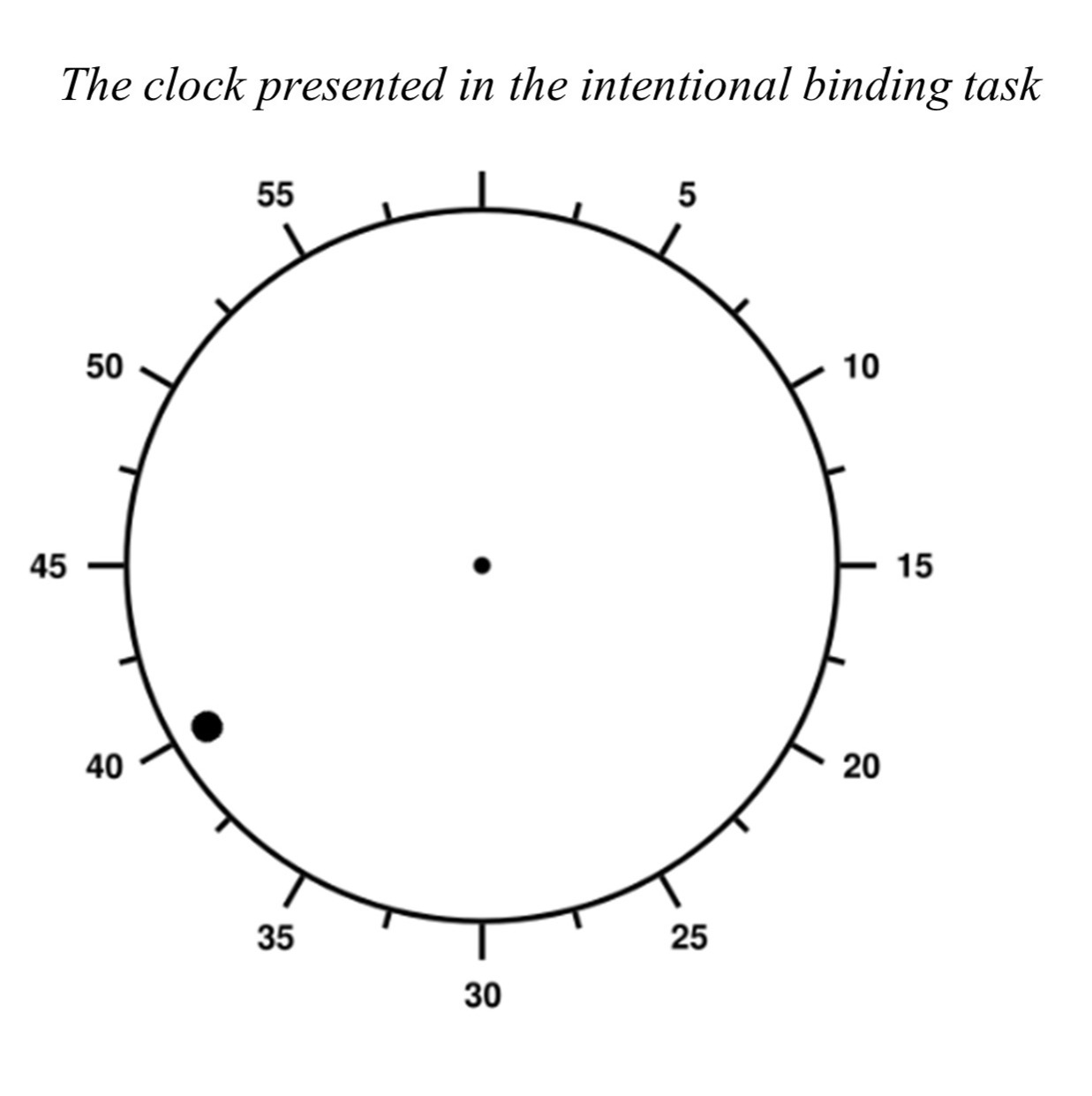

Supplement: S1 Fig — (TIF) [file pone.0341316.s008.tif]
