# Supplementary material for: Mirror-gazing-induced dissociation impairs self-reported and implicit sense of agency: A causal investigation of dissociation and agency under controlled laboratory conditions
Source: PLoS One. 2026 Feb 19;21(2):e0341316. doi: 10.1371/journal.pone.0341316 (PMC12919786; doi:10.1371/journal.pone.0341316)
Supplement: Attachment — Submitted filename: Response to Reviewers.docx [file pone.0341316.s010.docx]

# Response to Reviewers

I thank both reviewers for the attention they dedicated to this manuscript. Their comments highlighted areas in the text that were not clear enough, and I believe the revised version is noticeably improved. My answers to the specific comments are below.

## Reviewer #1:

The manuscript investigates the relevant question of the causal relationship between "dissociative detachments" and sense of agency, which has considerable clinical implications. It should be noted that, in their manuscript, the effect on agency is measured "after" the dissociative task, and not "during" dissociation. Conversely, this last question remains an open endeavor that still has to be investigated. The experimental results of the manuscript may prove interesting.

However, the authors should address important issues before their manuscript can be published. Issues concern both their technical/methodological errors and theoretical considerations.

Major issues:

1. Technical neglects include the lack of information about the illumination level of the face in front of the mirror. The authors do not report any data about the type of illuminator (LED, halogen, or tungsten, which have entirely different spectral distributions), the power of the lamp, and the face illuminance level. Conversely, facial illuminance is crucial for obtaining dissociative states and "dissociative detachments" during mirror-gazing. One can easily and inexpensively make these simple measurements through a low-cost photometer. The authors should report these data in their revision.

**Response:**

Thank you for the important technical comment. Designing the experimental procedure, we attempted to follow Caputo's rationale [1, 2] and therefore used a 25W incandescent bulb, as described in those articles. This is now mentioned in the Method section of Study 1 (lines 220-221). Additionally, following your comment, we now checked the illumination level of the face of the person sitting in the room under these conditions with a photometer. The result was between 3-5 lux. Although this is higher than the illumination level in Caputo’s studies, it suggests that higher lux levels may also produce dissociative responses, as reflected by the significant pre-post results of our two studies. Notably, studies other than Caputo’s have not reported lux levels; this is mentioned in a recent review [3]). We agree that reporting them better promotes clinical science; hence, we now describe our lux level in the Method section of Study 1 (lines 226-230) and also address it as a possible limitation in the General Discussion section (lines 643-646).

2. A second technical error concerns the setup of the laboratory (Figure 1). The placement of both the participant and the illuminator is totally asymmetrical. This situation is unsuitable for checking perceptual "dissociative detachments" since the participant cannot distinguish between biased perceptions of reality (produced by asymmetric facial illuminance) and the perception of dissociative faces that they can see in (or beyond) the mirror. The authors can overcome this issue in their future experiments by (1) placing the mirror in the exact geometrical center of the laboratory, (2) assuring that the wall reflected in the mirror (i.e., "behind" the participant) should be completely empty (which is not the case in the setup of Figure 1), (3) the illuminator lamp should be placed on the floor behind the participant and aligned on the symmetry axis of the laboratory, and (4) the facial illuminance should be about 1 lux or less when using a tungsten or halogen lamp. For instance, avoiding any suggestion or artifacts in facial illumination due to physical asymmetry in lighting is especially crucial when investigating brain self-generated dissociative detachments of chimeric faces through split-mirror gazing (Caputo, 2021, cited). However, suppose the authors aim to use a "more effective than the standard mirror-gazing paradigm" (line 593): in that case, the authors should first correct their errors, as indicated above, because the correct standard setup is much more effective than they ascertain in the two experiments of their manuscript.

**Response:**

Following this comment, we understood that we had not described the procedure in sufficient detail. We designed it with the intention of keeping the wall behind the participant empty. The lighting was placed on the floor and not on the wall. We now realize that this is not clear from a top-view illustration, and therefore we note it in the manuscript (lines 221-224). The lamp was not placed directly behind the participant, because in such a position it would have been reflected in the mirror. Therefore, it was placed in the back corner of the room. As we noted in response to the previous comment, we measured the intensity of the light illuminating the participant’s face, which was between 3-5 lux. We now present all these additional details more clearly in the manuscript.

Regarding the effectiveness of the paradigm, the sentence cited above (regarding the split mirror paradigm possibly being more effective) was erased. Additionally, we now address in more detail the differences between our procedure and Caputo's procedure in the limitations paragraph of the General Discussion (lines 643-646).

3. A third technical error concerns task instructions regarding the mirror-gazing paradigm. The participant should be asked to stare at their own eyes reflected in the mirror and not just vaguely gaze at their face.

**Response:**

We followed the instructions detailed in “Strange-face-in-the-mirror illusion” [2], in which the task was to gaze at the reflected face in the mirror using a 25W incandescent light bulb.

We debated whether to specifically instruct participants to focus on their eyes or to gaze more generally at their face. One article by Caputo from 2010 [1] specifically mentions the eyes, while the second, shorter article from the same year [2] does not mention this instruction, but rather refers more generally to gazing at one’s face as reflected in a mirror. We decided to adopt the less focused instruction, assuming that a focused instruction may hinder the formation of a dissociative experience [4]. In any case, we now mention this difference between our protocol and Caputo’s in Study 1 Method section (lines 234-236) and in the general discussion (line 645).

4. A fourth technical error is that the experimenter had no means to control for the correct execution of the mirror-gazing task. Indeed, "the participants sat alone in a dimly lit, private inner room" (line 205) with the "experimenter waiting in the larger room outside" (line 206). Therefore, the experimenter cannot control whether the participants directed their gaze correctly toward the mirror, at its frame, or away from the mirror. This impediment is a major drawback of the manuscript and may explain the tiny or absent difference between mirror-gazing and watching a naturalistic video in dissociation or absorption scores. The authors can overcome this crucial issue in future experiments by adopting the technical solution described in an article (Caputo, 2023, Journal Trauma Dissociation, not cited) by placing a small camera in the center of the mirror (with the face visible through approx 3 millimeter hole in the mirror) or just above the mirror.

**Response:**

In many psychological experiments, we rely on participants’ active participation, yet we do not monitor it, because our presence or the presence of a camera might influence the experimental procedure and will surely complicate it. For example, most studies using computerized tasks do not examine with a camera or eye-tracker whether participants followed the instruction to look at a target object. Such a procedure of including a camera in computerized tasks is not standard in the field of Psychology. We believe that the presence of a camera may affect participants’ responses to the paradigm, and perhaps even disrupt the dissociation induction process. Moreover, we do not think that not having an experimenter in the room should be necessarily considered as an error, as experimenter presence also has downsides (i.e., each approach has advantages and disadvantages). Experimenter presence for assuring the gaze direction of the participant is also quite subjective, and the constant gaze of the experimenter in charge of such a task towards the participant could interact with the experiences of the participant. In the present case, if participants did not look at the mirror at all, we would not expect any increase in the level of dissociation in any condition. Since the level of dissociation increased significantly pre- to post-manipulation, it seems that overall, participants cooperated with the task. The lack of a satisfactory difference between the experimental and control conditions most likely stems from the choice of an inappropriate control condition in Study 1.

5. These errors, which have been listed above, may have caused the low/absent dissociative states measured at T2 after the mirror-gazing task. The authors should declare these errors in a sub-chapter within the General Discussion of their main manuscript. This sub-chapter should be entitled "Limitations" and placed toward the end of their revised paper. These declarations might exclude (or reduce) the likelihood of persevering into similar flawed executions of the mirror-gazing procedure and setup.

**Response:**

The requested section was added to the limitations paragraph in the General Discussion (lines 643-646). We wish to note, however, that there were significant changes in pre- to post-mirror-gazing dissociation (and not low/absent). Notably, Caputo [2] himself, when describing the mirror gazing paradigm, noted that “the details of the setting (in particular the room illumination) are not critical.” This remark influenced our decision to use the Caputo procedure as a guidance, or a source of inspiration, and not aim for an immaculate replication. We now underscore that our protocol was not identical to Caputo’s but rather introduced some adaptations (lines 104-110).

6. The face conveys one's identity. Hence, mirror-gazing certainly involves both face perception and identity recognition. Therefore, limiting the effect of prolonged mirror gazing only to "dissociative detachments" is incorrect. A famous paper on dissociative states (Holmes et al, 2005, Clinical Psychology Review, not cited) distinguished "dissociative detachments" and "compartmentalizations" of other identities. The authors should update and correct their incomplete theoretical approach to mirror-gazing.

**Response:**

We did not elaborate on the conceptualization of dissociative detachments and compartmentalizations [5], as we sensed that it is not an essential notion for this investigation. Originally, we had mentioned briefly (only in the abstract) that the observed phenomena in our experiments were dissociative detachments. Following this comment, however, we deleted this definition from our abstract, as the abstract should not contain information that is not discussed in the manuscript itself.

Still, we see fit to respond here regarding how we see Holmes’ et al.’s conceptualization in the context of our study. Detachments, according to Holmes, include separation of the self from certain aspects of everyday experience. Compartmentalizations, on the other hand, are characterized by difficulty controlling actions, or cognitive processes, which are usually voluntarily controlled. The most prominent aspect of compartmentalization is that the compartmentalized processes continue, although being inaccessible, and their intact flow affects emotion, thoughts and actions. In other words, the main difference between detachments and compartmentalizations according to Holmes et al. is the presence of “split” functions that operate independently. Holmes and colleagues emphasize that detachments and compartmentalizations are usually distinguished and do not occur together. As we understand their conceptualization, the mirror-gazing paradigm causes a detachment from a certain aspect of everyday life, specifically, the familiar appearance of oneself. Although the paradigm certainly influences aspects of identity experience, we do not recognize that it generates a compartmentalized process that continues to operate independently and autonomously. Therefore, we believe that the experience it evokes cannot be classified as compartmentalization.

7. The measure (CADSS) used by the authors for DPDR is inadequate when evaluating mirror-gazing dissociation since it contains no items concerning mirrors. At the same time, DES has only one item dedicated to mirror gazing, and this item could explain the effect of "trait dissociation" that the authors found with various statistical interactions, given that DES (i.e., measurement of trait dissociation) was administered "after" the dissociative task (line 307). In the MGS group, the "suggestion" of paying attention to the face probably did not introduce a bias, as the authors suppose. Instead, it may enhance awareness of face changes in participants who, as a consequence, scored higher on DES.

**Response:**

As we see it, to establish that the clinical construct termed “dissociation,” harboring the widely-accepted meanings and definitions of this label, is indeed the outcome induced by mirror-gazing, it should be measured using its validated and well-known general scales, without necessarily asking specifically about a reflection in the mirror. Using a general validated state assessment tool for dissociation supports the convergent validity of the measured construct and shows that the resulting phenomenon is broader than momentary perceptual distortions regarding reflection, encompassing the broader aspects of the dissociative experience.

Regarding the mirror item in the DES, we thank the reviewer for this important observation, whereby results we considered as “trait” could have been affected by state influences on the mirror item. Following this observation, we conducted again all analyses involving the DES while excluding the mirror item, which may have been affected by the manipulation. Results remained unchanged. This is now mentioned in the Results sections of Study 1 and Study 2 (lines 296-301, 475-475).

8. Supplementary materials:
   The authors only mention early explanations of mirror-gazing "dissociative detachments" and omit more recent theoretical accounts. It should be noted that these "dissociative detachments" are specifically linked to face stimuli, thus leading to identity discontinuities since the face affords one's identity. Therefore, from the very beginning, these "dissociative detachments" of one's face can exclude the simple explanation based on the Troxler effect. All three dissociative states (i.e., derealization, depersonalization, and dissociative identity) may be involved in "dissociative detachments" [and compartmentalization of new strange identities]. Indeed, Caputo (2023, Journal Trauma Dissociation, not cited) adopted this theoretical perspective and found that the CADSS scale is not an optimal scale for measuring mirror-gazing "dissociative detachments" [and identity compartmentalization] (indeed, this CADSS inadequacy is confirmed by the description of unsettling experiences of mirror gazing by one participant: lines 290-296).

**Response:**

Thank you for the important clarification regarding the theoretical explanations of the strange face illusion included in the supplementary materials. We have now added newer perspectives and the missing citations to the relevant section (which is titled now “S1”) describing the scientific understanding of the mechanisms governing the effect.

Regarding the comment about measurement: since the current investigation does not focus on the strange-face illusion itself but rather on its psychological impact, and specifically, on the increase in dissociative states that follows it, we considered it important to use a dedicated measure of dissociation, such as the CADSS. However, it is certainly possible that we would have benefited from a dedicated measure of the illusion as well. Using the SFQ could have helped us better organize and understand what our participants experienced during the experiment. We now address this in our limitations paragraph (lines 646-649).

9. Finally, the latest structural equation model that investigates "dissociative detachments" [and identity compartmentalization] (Lange et al, 2021, Psychology of Consciousness, not cited) found empirical evidence that the three layers of dissociative states (i.e., 1. derealization, 2. depersonalization, and 3. dissociative identity) are linked together in that exact sequence by causal relationships during eye-to-eye gazing, i.e., the inter-personal version of mirror-gazing of oneself.

**Response:**

Thank you, this model was added to the review of the strange face illusion underlying mechanisms included in the supporting information files (see S1).

10. Minor issues:
    Responses to CADSS items are not on Likert scales but on 5-level scales.

**Response:**

The term “Likert” was deleted from the description of the CADSS.

## Reviewer #2:

1. INTRODUCTION

In the introduction section, the authors briefly describe explicit and implicit measures of sense of agency (lines 79-86). I suggest expanding the description of the experimental paradigm previously employed to implicitly assess the sense of agency. Specifically, the authors refer only to the intentional binding paradigm, but they could also describe alternative paradigms such as the sensory attenuation paradigm, which has been successfully employed to investigate alterations of sense of agency also in Borderline Personality Disorder, in which patients often manifest dissociation symptoms (see for examples: <https://doi.org/10.3389/fpsyt.2020.00449>, <https://doi.org/10.1016/j.neuroimage.2021.117727>, <https://doi.org/10.1523/JNEUROSCI.1694-16.2016>, <https://doi.org/10.17605/OSF.IO/SZTEK>, <https://doi.org/10.1111/ejn.13931>, <https://doi.org/10.1038/s41598-023-41345-5>).

**Response:**

Following this comment, we now also mention the sensory attenuation paradigm in the introduction (lines 86-94).

2. STUDY 1 RESULTS section
   I suggest moving Table 1 to the supplementary materials and replace it with a meaningful graphical representation of the significant interactions emerging from the mixed ANCOVAs on Manipulation check and Self-reported sense of agency. Furthermore, even if full details of post-hoc analyses are provided in the supplementary materials, when describing the results of Bonferroni-Holm post-hoc test, authors should also report the p-values, at least those of significant comparisons. It would help the reader to have an immediate grasp of the statistical significance, thereby increasing the strength of the results.

**Response:**

This was done. All the results’ tables were moved to the supporting information files (see S2, S3, S7, S8). Line charts (Figures 2, 3, 4) depicting the manipulation check and self-reported sense of agency ANCOVAs were added to the Results section of Study 1. The p-values of the post-hoc tests are provided now in the manuscript itself.

3. STUDY 2 RESULTS section
   As for the study 1, I again suggest moving Table 2 to the supplementary materials and replace it with a meaningful graphical representation of the significant results of manipulation check, self-reported sense of agency and intentional binding. Even in this case, please provide p-values of significant post-hoc comparisons.

**Response:**

Again, this was done. Table 2 was moved to the supporting information files . We added to the Results section of Study 2, line charts describing the manipulation check ANCOVAs and the self-reported agency ANCOVA (Figures 5, 6, 7), and a bar chart describing the planned comparisons of the agency judgment error between the different study groups (Figure 8). We also report now in the manuscript the p-values of the post-hoc comparisons.

4. GENERAL DISCUSSION
   In the general discussion, authors discussed clinical implications of their work, reporting the cases of Schizophrenia and OCS as related to impaired sense of agency. I suggest expanding this part by also mentioning other psychiatric conditions in which dissociative symptoms are experienced, such as Borderline Personality Disorder and Eating Disorders with a bulimic variant, where alterations of sense of agency have also been previously described.

**Response:**

Thank you, this was done. We now also discuss the contributions of our findings to the understanding of BPD and bulimia nervosa, and mention recent relevant articles (lines 580-588).

# References:

1. Caputo GB. Apparitional experiences of new faces and dissociation of self-identity during mirror gazing. Percept Mot Skills. 2010;110(3):1125–38.
2. Caputo GB. Strange-face-in-the-mirror illusion. Perception. 2010;39(7):1007–8.
3. Mash J, Jenkinson PM, Dean CE, Laws KR. Strange face illusions: A systematic review and quality analysis. Conscious Cogn. 2023;109:103480. <https://doi.org/10.1016/j.concog.2023.103480>
4. Morgan CA 3rd, Taylor MK. Spontaneous and deliberate dissociative states in military personnel: are such states helpful? J Trauma Stress. 2013;26(4):492–7.
5. Holmes EA, Brown RJ, Mansell W, Fearon RP, Hunter EC, Frasquilho F, Oakley DA. Are there two qualitatively distinct forms of dissociation? A review and some clinical implications. Clin Psychol Rev. 2005;25(1):1–23.
