# Supplementary material for: Mirror-gazing-induced dissociation impairs self-reported and implicit sense of agency: A causal investigation of dissociation and agency under controlled laboratory conditions
Source: PLoS One. 2026 Feb 19;21(2):e0341316. doi: 10.1371/journal.pone.0341316 (PMC12919786; doi:10.1371/journal.pone.0341316)
Supplement: Attachment — Submitted filename: Response to Reviewers R2.docx [file pone.0341316.s011.docx]

# Response to Reviewers

I thank reviewer #1 for their comments. I have revised the manuscript accordingly, and my detailed responses are provided below.

## Reviewer #1:

1. The authors do not mention the main limitation of their work: the complete absence of controls during the participants' correct execution of the experimental task. This drawback is particularly critical in the mirror-gazing task because this task can produce unsettling experiences in some participants (see the typical experience of depersonalization/derealization that is reported in lines 325-331). In their response, the authors gave a weak justification for their neglect, given that the presence of a micro-camera across all experimental conditions produced a similar effect across all experimental conditions, offering the opportunity to monitor the participant without the need for an experimenter in the laboratory. Conversely, monitoring of correct gazing through a video-camera can be very effective in mirror-gazing tasks: see standard measures of dissociation in Caputo (2023, Journal Trauma Dissociation, not cited).
   The authors should include this limitation in the final section of the discussion. It is important to acknowledge this limitation, as future studies (possibly by the authors themselves) should keep this in mind.

**Response:**

The requested limitation was added to the limitations paragraph (lines 658-661).

2. Line 31. "dissociative absorption" - This is an incorrect definition that creates confusion: normal absorption is not a dysfunctional dissociative state. (Dalenberg, C. J., Katz, R. R., Thompson, K. J., & Paulson, K. (2022). The case for the study of “normal” dissociation processes. In: Martin J. Dorahy, Steven N. Gold, John A. O’Neil (eds.), Dissociation and the dissociative disorders (pp. 81-92), not cited). The authors should not insist in this error concerning dissociation that is still present in the literature.

**Response:**

We believe that our approach completely aligns with the chapter referenced in this comment, as the authors of that chapter conclude that absorption is part of the dissociative domain. For example, in their concluding remarks, their first conclusion is: "Empirical evidence, the evidence that should ultimately guide us, suggests that "normal" dissociation, or absorption, is a form of dissociation". (pp. 89 in Dalenberg et al., The case for the study of "normal" dissociation processes, In: Dorahy et al., 2022, Dissociation and the Dissociative Disorders). However, as the term "dissociative absorption" bothered the reviewer, we changed it. In the previous version of the manuscript, it was only used once - in the abstract ("...but the control condition was not sufficiently differentiated, also increasing dissociative absorption"). In the new revised version of the manuscript, we changed to the following:

"...but the control condition was not sufficiently differentiated, as it also increased absorption, a construct which is considered as part of the domain of dissociative experiences".

3. Lines 112-115. "As we read the Caputo [26] procedure, we noticed that participants were informed in advance that they might notice perceptual changes in their reflections. We speculated that such a warning might have influenced Caputo’s results as it contained a potentially suggestive cue." This is an outdated, ill-posed question to which the article mentioned (Caputo, 2023) has already responded. This ill-posed question concerning supposedly the effects of the "suggestion" is not correct. Indeed, these effects are simply caused by differences in working memory storage, an issue common to many cognitive and perceptual behaviors when the task for participants to perform is lacking, yielding no memory traces. It is not caused by the real existence of changes to the level of dissociation by the mirror-gazing task, but only by the absence of a task to encourage participants in the MG condition (compared to MGS condition). In fact, the level of dissociation can remain the same, although a difference is detected by self-report questionnaires of dissociation that are based on conscious memory.

**Response:**

Following this comment, we have refined our approach to the subject of suggestion. First, because our data collection was conducted before the publication of the 2023 research, we were not familiar with that article when designing the present study. Second, we believe that our findings contribute to existing knowledge, as we directly compared, within the same research design and sample, participants who received suggestive instructions with those who received more general instructions. Furthermore, our findings align with the 2023 results. We now note this both toward the end of the Introduction section (lines 120-129) and in the Discussion of Study 2 (lines 569-571 ).
